# Sotrovimab in Hospitalized Patients with SARS-CoV-2 Omicron Variant Infection: a Propensity Score-Matched Retrospective Cohort Study

**DOI:** 10.1128/spectrum.04103-22

**Published:** 2022-12-08

**Authors:** Marcel S. Woo, Thomas Theo Brehm, Marlene Fischer, Andreas Heyer, Dominic Wichmann, Sabine Jordan, Dominik Nörz, Marc Lütgehetmann, Marylyn M. Addo, Ansgar W. Lohse, Stefan Schmiedel, Stefan Kluge, Julian Schulze zur Wiesch

**Affiliations:** a Department of Neurology, University Medical Center Hamburg-Eppendorf, Hamburg, Germany; b Institute of Neuroimmunology and Multiple Sclerosis (INIMS), Center for Molecular Neurobiology Hamburg (ZMNH), University Medical Center Hamburg-Eppendorf, Hamburg, Germany; c I. Department of Medicine, University Medical Center Hamburg-Eppendorf, Hamburg, Germany; d German Center for Infection Research (DZIF), University Medical Center Hamburg-Eppendorf, Lübeck-Borstel-Riems, Hamburg, Germany; e Department of Intensive Care Medicine, University Medical Center Hamburg-Eppendorf, Hamburg, Germany; f Institute of Medical Microbiology, Virology and Hygiene, University Medical Center Hamburg-Eppendorf, Hamburg, Germany; g Institute of Infection Research and Vaccine Development (IIRVD), University Medical Center Hamburg-Eppendorf, Hamburg, Germany; University of Mississippi Medical Center

**Keywords:** SARS-CoV-2, COVID-19, Omicron, sotrovimab, remdesivir, monoclonal antibodies, Omicron variant

## Abstract

*In vitro* data suggest the monoclonal antibody sotrovimab may have lost inhibitory capability against the severe acute respiratory syndrome coronavirus 2 (SARS-CoV-2) Omicron variant. We aimed to provide real-life data on clinical outcomes in hospitalized patients. We retrospectively analyzed patients who were treated at the University Medical Center Hamburg-Eppendorf, Germany, between December 2021 and June 2022. Out of all 1,254 patients, 185 were treated with sotrovimab: 147 patients received sotrovimab monotherapy, and 38 received combination treatment with sotrovimab and remdesivir. We compared in-hospital mortality for the different treatment regimens for patients treated on regular wards and the intensive care unit separately and performed propensity score matching by age, sex, comorbidities, immunosuppression, and additional dexamethasone treatment to select patients who did not receive antiviral treatment for comparison. No difference in in-hospital mortality was observed between any of the treatment groups and the respective control groups. These findings underline that sotrovimab adds no clinical benefit for hospitalized patients with SARS-CoV-2 Omicron variant infections.

**IMPORTANCE** This study shows that among hospitalized patients with SARS-CoV-2 Omicron variant infection at risk of disease progression, treatment with sotrovimab alone or in combination with remdesivir did not decrease in-hospital mortality. These real-world clinical findings in combination with previous *in vitro* data about lacking neutralizing activity of sotrovimab against SARS-CoV-2 Omicron variant do not support sotrovimab as a treatment option in these patients.

## INTRODUCTION

As of September 2022, more than 6.4 million people have died from coronavirus disease 2019 (COVID-19) worldwide (1). The severe acute respiratory syndrome coronavirus type 2 (SARS-CoV-2) Omicron variant (B.1.1.529) emerged in November 2021 and quickly replaced the Delta variant (B.1.617.2) as the predominant cause of COVID-19 globally. The high number of mutations observed in the spike protein is responsible for immune evasion, including breakthrough infections among fully vaccinated individuals (2). Also, most anti-SARS-CoV-2 monoclonal antibodies (MABs) in clinical use were shown to be ineffective against the Omicron variant (3). Sotrovimab is a recombinant engineered human monoclonal IgG1 antibody that binds to a highly conserved epitope on the spike protein receptor binding domain of SARS-CoV-2. It shows neutralizing activity against the Omicron BA.1 sublineage but not the BA.2, BA.4, and BA.5 sublineages (3–6). It was granted marketing authorization by the European Commission in December 2021 for the treatment of adults and adolescents aged >12 years and weighing more than 40 kg who suffer from COVID-19, do not need oxygen supplementation, and have an increased risk of developing a severe disease course (7). Sotrovimab has been shown to reduce the risk of disease progression among nonhospitalized high-risk patients with mild to moderate COVID-19 when ancestral-like SARS-CoV-2 variants were predominant (8, 9). Surprisingly, data on the clinical efficacy of sotrovimab in hospitalized patients with Omicron variant infections are scarce. At the University Medical Center Hamburg-Eppendorf, we previously described clinical characteristics and outcomes of hospitalized patients with COVID-19 during the earlier phases of the pandemic (10, 11). Here, we conducted a single-center retrospective propensity score analysis of patients hospitalized with SARS-CoV-2 Omicron variant infections.

## RESULTS

**FIG 1:**
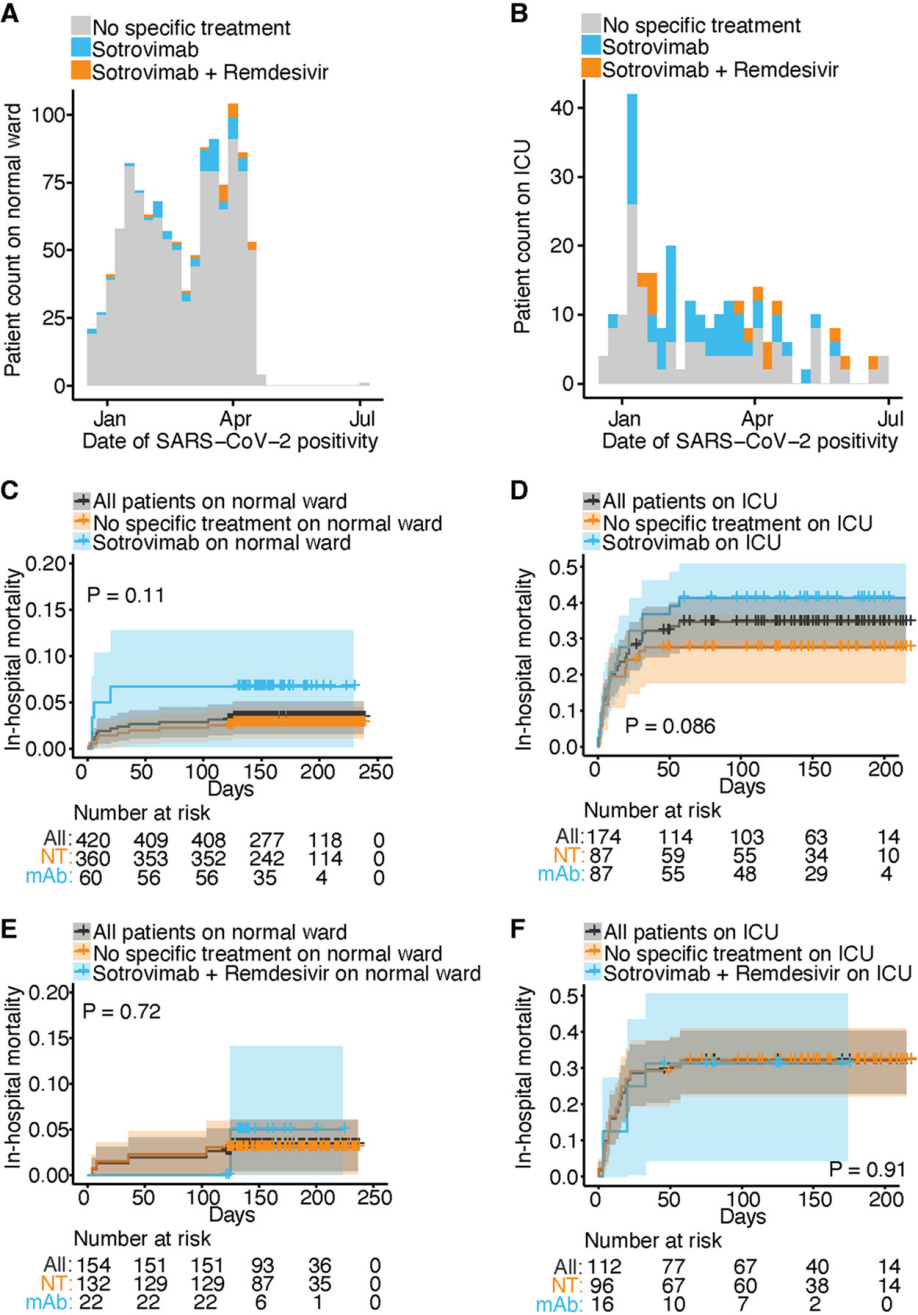
In-hospital mortality in hospitalized patients with SARS-CoV-2 Omicron variant infections. (A and B) Patients included in the analysis who were treated in a normal ward (A) and in the intensive care unit (B) by date of SARS-CoV-2 infection. In-hospital mortality is shown for the following treatment groups and respective propensity score-matched control groups who did not receive antiviral treatment (NT): patients who were treated with sotrovimab alone in a normal ward (C), patients who were treated with sotrovimab alone in the intensive care unit (D), patients who were treated with combination therapy with sotrovimab and remdesivir in a normal ward (E), and patients who were treated with combination therapy with sotrovimab and remdesivir in the intensive care unit (F). “Number at risk” refers to the number of patients who were still hospitalized at the respective time after SARS-CoV-2 infection.

During the study period, a total of 1,254 patients with SARS-CoV-2 Omicron variant infections were admitted and treated at the University Medical Center Hamburg-Eppendorf. Of those, 147 were treated with sotrovimab alone, and 38 received combination treatment with sotrovimab and remdesivir. After propensity score matching was performed, patients in the different treatment subgroups and the respective control groups did not differ in terms of sex, age, Charlson comorbidity index (CCI), treatment with dexamethasone, vaccination status, and history of coronary heart disease, chronic pulmonary disease, diabetes mellitus with and without end organ damage, and chronic kidney disease (see Tables S1 to S4 in the supplemental material). The rate of patients with congestive heart failure was lower in the treatment group than in the control group for patients who received monotherapy with sotrovimab in the intensive care unit (ICU) group (67.8% [*n* = 59] versus 85.1% [*n* = 74]; *P* = 0.012) and did not differ between the remaining treatment and control groups. Patients treated with sotrovimab as monotherapy on the regular ward less frequently received immunosuppressive therapy than the control group (61.7% [*n* = 37] versus 77.2% [*n* = 278]; *P* = 0.016), but the rate of patients with immunosuppressive therapy did not differ between the other treatment and propensity score-matched control groups. Also, patients treated with sotrovimab as monotherapy in the ICU less frequently received chemotherapy than the control group (77.0% [*n* = 67] versus 100% [*n* = 87]; *P* < 0.001). Among other subgroups, the rates of patients receiving chemotherapy did not differ between treatment and control groups. Among patients who were treated in a normal ward, rates of in-hospital mortality did not differ between treatment and control groups for those who were treated with sotrovimab (6.7% [*n* = 4] versus 2.8% [*n* = 10]; *P* = 0.11), and for those who were treated with sotrovimab and remdesivir (4.5% [*n* = 1] versus 3.0% [*n* = 4]; *P* = 0.72) (Fig. 1). Likewise, no difference in the rates of in-hospital mortality was observed between ICU patients and those treated with sotrovimab alone (41.4% [*n* = 36] versus 27.6% [*n* = 24]; *P* = 0.09), or in combination with remdesivir (31.2% [*n* = 5] versus 32.3% [*n* = 31]; *P* = 0.91) and the respective control groups. In addition, we compared rates of in-hospital mortality for patients with Omicron BA.1, BA.2, or BA.4/5 sublineage infections for all subgroups and observed no significant differences (Table S1).

## DISCUSSION

This propensity score-matched retrospective cohort study demonstrates that sotrovimab is not effective in the treatment of hospitalized patients with SARS-CoV-2 Omicron variant infections. It was recently shown that in high-risk outpatients with mainly SARS-CoV-2 Omicron BA.1 sublineage infections, early treatment with sotrovimab significantly lowers the risk of hospitalization (12). However, *in vitro* data have shown that sotrovimab appears to have retained activity against the Omicron BA.1 sublineage, but it has lost inhibitory capability against the BA.2, BA.4, and BA.5 sublineages (3–6). In our cohort, no significant difference in rates of in-hospital mortality was observed among patients infected with different SARS-CoV-2 Omicron sublineages in the different treatment cohorts. While the patient numbers in subgroups may not be sufficient to show differences in clinical outcomes among patients infected with different Omicron sublineages, the lack of clinical benefit of sotrovimab administration in the overall cohort suggests that it is not effective in the treatment of hospitalized patients infected with the currently circulating SARS-CoV-2 sublineages BA.4 and BA.5. Remdesivir is a direct-acting nucleotide inhibitor of SARS-CoV-2 RNA-dependent RNA polymerase that was first approved by the European Medicines Agency (EMA) in June 2020 (13). Recent data show that high-risk outpatients with Omicron BA.1 infections who receive early treatment with remdesivir are less likely to be hospitalized (12). Remdesivir has recently been shown to have equipotent antiviral activity against the different SARS-CoV-2 variants, including the different Omicron sublineages (5, 14). The fact that combination treatment with sotrovimab and remdesivir did not result in improved clinical outcomes in our study cohort suggests that delayed antiviral administration in hospitalized patients’ treatment is generally not effective. Administration of sotrovimab was only recommended in high-risk patients 5 to 7 days after onset of symptoms and not in those who have been hospitalized due to COVID-19. Our data suggest that sotrovimab does not affect in-hospital mortality in hospitalized patients with SARS-CoV-2 Omicron variant infections. We were not able to determine whether this is a result of the lack of neutralizing activity of sotrovimab against the Omicron variant or whether MAb administration is generally ineffective in reducing hospital mortality among patients already hospitalized with Omicron infections. Our study is subject to several limitations inherent to the single-center retrospective study design that should be noted. Since no regular follow-up after hospital discharge was performed, only in-hospital mortality but not overall survival could be investigated. Furthermore, our monocentric study design warranted a limited number of patients. Vaccination status was not available for all patients, so we were not able to reliably adjust for this potential confounding factor in all individuals. Of note, as of April 2022 the WHO recommends against the treatment with sotrovimab due to the reduced neutralization activity *in vitro* of the currently circulating variants (15). Despite these limitations, our study offers considerable value as one of the first real-world studies to investigate the impact of treatment with sotrovimab in hospitalized patients with SARS-CoV-2 Omicron variant infections. Future studies are needed to investigate the association between treatment with novel MAbs and clinical outcomes in patients infected with SARS-CoV-2 Omicron sublineages or novel variants and to explore the factors involved in the effectiveness of the antiviral medication.

### Conclusion.

Among hospitalized patients with SARS-CoV-2 Omicron variant infection at risk of disease progression, treatment with sotrovimab alone or in combination with remdesivir did not decrease in-hospital mortality. These real-world clinical findings in combination with previous *in vitro* data about the lack of neutralizing activity of sotrovimab against the SARS-CoV-2 Omicron variant do not support sotrovimab as a treatment option in these patients.

## MATERIALS AND METHODS

We retrospectively identified all hospitalized patients with SARS-CoV-2 Omicron variant infections who received sotrovimab alone or in combination with remdesivir at the University Medical Center Hamburg-Eppendorf between December 2021 and June 2022. The study was reviewed and approved by the Ethics Committee of the Medical Council of Hamburg (WF-052/20). SARS-CoV-2 variant of concern (VOC) typing was part of the routine virological diagnostic, and samples were analyzed in an accredited diagnostic lab. VOC typing for the different linages was performed using several high-throughput TaqMan-based quantitative PCR (qPCR) panels based on detection of multiple mutations in the viral spike protein as previously published (16–18). Briefly, for Omicron VOC typing the qPCR detects E484A, A67V plus del-HV69-70, and N679K plus P681H. Therefore, the assay allows for differentiation between BA.1 and BA.2 and with modified cutoff relative fluorescence increase RFI values (BA.4/5 is missing the BA.1 typical A67V mutation) also for BA.4/5. All qPCR typing assays were validated and monitored against SARS CoV-2 whole-genome data generated by next-generation sequencing. We compared the rate of in-hospital mortality for patients who received antiviral treatment with that of a control group of patients with SARS-CoV-2 Omicron variant infections who did not receive antiviral treatment. To minimize confounding from sex, age, Charlson comorbidity index (CCI), immunosuppression, treatment with dexamethasone, and vaccination status, a propensity score was generated for each patient. Patients in the respective treatment group were matched to the patient who did not receive antiviral treatment at a 1:6 ratio for patients treated in the regular ward and at a 1:1 ratio for patients treated in the ICU using a nearest-neighbor matching algorithm. This comparison was performed for those patients treated on regular wards and for those treated on the intensive care unit (ICU) separately to account for disease severity. Statistical analyses were carried out within the R environment (version 1.2.5.002) on a Mac OS X. For categorical variables, the chi-square test or Fisher exact test was used. For continuous variables, Student's *t* test was used. A *P* value of <0.05 was determined to indicate statistical significance.

### Ethics approval and consent to participate.

This study was reviewed and approved by the Ethics Committee of the Medical Council of Hamburg (WF-052/20).

### Data availability.

All data generated or analyzed during this study are included in this article.
