# Supplementary material for: Sotrovimab in Hospitalized Patients with SARS-CoV-2 Omicron Variant Infection: a Propensity Score-Matched Retrospective Cohort Study
Source: Microbiol Spectr. 2022 Dec 8;11(1):e04103-22. doi: 10.1128/spectrum.04103-22 (PMC9927465; doi:10.1128/spectrum.04103-22)
Supplement: Supplemental file 1 — Supplemental material. Download spectrum.04103-22-s0001.pdf, PDF file, 0.8 MB [file spectrum.04103-22-s0001.pdf]

Supplemental Table 1. Patients treated with sotrovimab in the regular ward and propensity-matched control group

		**Control group**	**Treatment group**	**P**
Sex	female	124 (34.4)	20 (33.3)	0,983
	male	236 (65.6)	40 (66.7)	
Age	Mean (SD)	51.2 (22.1)	51.0 (21.3)	0,945
Vaccination status	≥ 2 vaccine doses	181 (50.3)	24 (40.0)	0,316
	< 2 vaccine doses	75 (20.8)	14 (23.3)	
	Unknown	104 (28.9)	22 (36.7)	
Dexamethason treatment	Yes	318 (88.3)	50 (83.3)	0,38
	No	42 (11.7)	10 (16.7)	
Charlson Comorbidity Index	Mean (SD)	3.2 (3.2)	3.3 (2.8)	0,88
Coronary heart disease	Yes	299 (83.1)	48 (80.0)	0,693
	No	61 (16.9)	12 (20.0)	
Chronic heart insufficiency	Yes	321 (89.2)	52 (86.7)	0,728
	No	39 (10.8)	8 (13.3)	
Chronic pulmonary disease	Yes	320 (88.9)	52 (86.7)	0,778
	No	40 (11.1)	8 (13.3)	
Diabetes mellitus without organ damage	Yes	325 (90.3)	57 (95.0)	0,349
	No	35 (9.7)	3 (5.0)	
Diabetes mellitus with organ damage	Yes	349 (96.9)	58 (96.7)	1
	No	11 (3.1)	2 (3.3)	
Chronic kidney disease	Yes	308 (85.6)	47 (78.3)	0,215
	No	52 (14.4)	13 (21.7)	
Chemotherapy	Yes	327 (90.8)	49 (81.7)	0,055
	No	33 (9.2)	11 (18.3)	
Immunosuppressive therapy	Yes	278 (77.2)	37 (61.7)	0,016
	No	82 (22.8)	23 (38.3)	

Supplemental Table 2. Patients treated with remdesivir and sotrovimab in the regular ward and propensity-matched control group

		**Control group**	**Treatment group**	**P**
Sex	female	77 (58.3)	12 (54.5)	0,92
	male	55 (41.7)	10 (45.5)	
Age	Mean (SD)	59.1 (17.3)	58.9 (16.4)	0,961
Vaccination status	≥ 2 vaccine doses	99 (75.0)	13 (59.1)	0,154
	< 2 vaccine doses	24 (18.2)	5 (22.7)	
	Unknown	9 (6.8)	4 (18.2)	
Dexamethason treatment	Yes	117 (88.6)	17 (77.3)	0,26
	No	15 (11.4)	5 (22.7)	
Charlson Comorbidity Index	Mean (SD)	3.8 (3.0)	4.1 (3.1)	0,705
Coronary heart disease	Yes	109 (82.6)	19 (86.4)	0,895
	No	23 (17.4)	3 (13.6)	
Chronic heart insufficiency	Yes	120 (90.9)	20 (90.9)	1
	No	12 (9.1)	2 (9.1)	
Chronic pulmonary disease	Yes	113 (85.6)	20 (90.9)	0,737
	No	19 (14.4)	2 (9.1)	
Diabetes mellitus without organ damage	Yes	116 (87.9)	20 (90.9)	0,959
	No	16 (12.1)	2 (9.1)	
Diabetes mellitus with organ damage	Yes	128 (97.0)	21 (95.5)	1
	No	4 (3.0)	1 (4.5)	
Chronic kidney disease	Yes	106 (80.3)	20 (90.9)	0,37
	No	26 (19.7)	2 (9.1)	
Chemotherapy	Yes	118 (89.4)	16 (72.7)	0,07
	No	14 (10.6)	6 (27.3)	
Immunosuppressive therapy	Yes	103 (78.0)	17 (77.3)	1
	No	29 (22.0)	5 (22.7)	

Supplemental Table 3. Patients treated with sotrovimab in the intensive care unit and propensity-matched control group

		**Control group**	**Treatment group**	**P**
Sex	female	33 (37.9)	37 (42.5)	0,643
	male	54 (62.1)	50 (57.5)	
Age	Mean (SD)	64.4 (14.3)	63.9 (22.2)	0,849
Vaccination status	Full	65 (74.7)	59 (67.8)	0,059
	Not full	14 (16.1)	25 (28.7)	
	Unknown	8 (9.2)	3 (3.4)	
Dexamethason treatment	Yes	32 (36.8)	20 (23.0)	0,068
	No	55 (63.2)	67 (77.0)	
Charlson Comorbidity Index	Mean (SD)	5.5 (3.2)	5.9 (3.9)	0,394
Coronary heart disease	Yes	63 (72.4)	66 (75.9)	0,729
	No	24 (27.6)	21 (24.1)	
Chronic heart insufficiency	Yes	74 (85.1)	59 (67.8)	0,012
	No	13 (14.9)	28 (32.2)	
Chronic pulmonary disease	Yes	66 (75.9)	70 (80.5)	0,582
	No	21 (24.1)	17 (19.5)	
Diabetes mellitus without organ damage	Yes	79 (90.8)	73 (83.9)	0,254
	No	8 (9.2)	14 (16.1)	
Diabetes mellitus with organ damage	Yes	77 (88.5)	81 (93.1)	0,431
	No	10 (11.5)	6 (6.9)	
Chronic kidney disease	Yes	63 (72.4)	63 (72.4)	1
	No	24 (27.6)	24 (27.6)	
Chemotherapy	Yes	87 (100.0)	67 (77.0)	<0.001
	No	0 (0.0)	20 (23.0)	
Immunosuppressive therapy	Yes	61 (70.1)	50 (57.5)	0,115
	No	26 (29.9)	37 (42.5)	

Supplemental Table 4. Patients treated with remdesivir and sotrovimab in the intensive care unit and propensity-matched control group

		**Control group**	**Treatment group**	**P**
Sex	female	32 (33.3)	5 (31.2)	1
	male	64 (66.7)	11 (68.8)	
Age	Mean (SD)	58.0 (19.6)	60.8 (16.5)	0,594
Vaccination status	≥ 2 vaccine doses	78 (81.2)	14 (87.5)	0,798
	< 2 vaccine doses	17 (17.7)	2 (12.5)	
	Unknown	1 (1.0)		
Dexamethason treatment	Yes	21 (21.9)	2 (12.5)	0,599
	No	75 (78.1)	14 (87.5)	
Charlson Comorbidity Index	Mean (SD)	4.9 (3.5)	5.1 (3.6)	0,843
Coronary heart disease	Yes	79 (82.3)	11 (68.8)	0,356
	No	17 (17.7)	5 (31.2)	
Chronic heart insufficiency	Yes	77 (80.2)	12 (75.0)	0,886
	No	19 (19.8)	4 (25.0)	
Chronic pulmonary disease	Yes	69 (71.9)	10 (62.5)	0,642
	No	27 (28.1)	6 (37.5)	
Diabetes mellitus without organ damage	Yes	87 (90.6)	15 (93.8)	1
	No	9 (9.4)	1 (6.2)	
Diabetes mellitus with organ damage	Yes	90 (93.8)	14 (87.5)	0,708
	No	6 (6.2)	2 (12.5)	
Chronic kidney disease	Yes	69 (71.9)	9 (56.2)	0,335
	No	27 (28.1)	7 (43.8)	
Chemotherapy	Yes	89 (92.7)	16 (100.0)	0,577
	No	7 (7.3)	0 (0.0)	
Immunosuppressive therapy	Yes	55 (57.3)	6 (37.5)	0,23
	No	41 (42.7)	10 (62.5)	

Supplemental Figure 1. In-hospital mortality in hospitalized patients with different SARS-CoV-2 Omicron sublineage infections

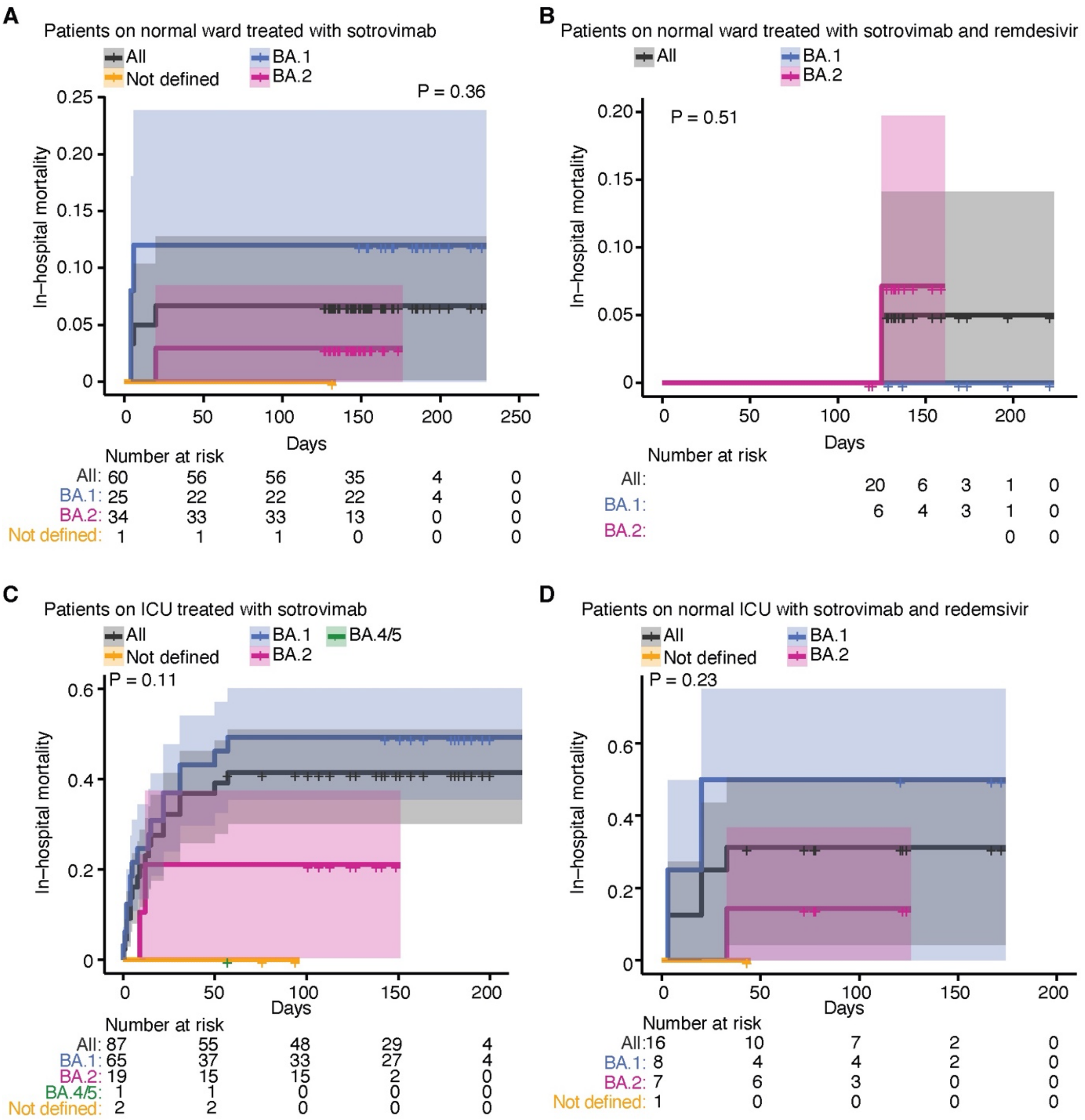

In-hospital mortality is shown for the following treatment groups and compared between SARS-CoV-2 Omicron BA.1, BA.2, and BA.4/5 sublineage infections: patients who were treated with sotrovimab alone in a normal ward (C), patients who were treated with sotrovimab alone in the intensive care unit (D), patients who were treated with combination therapy with sotrovimab and remdesivir in a normal ward (E), and patients who were treated with combination therapy with sotrovimab and remdesivir on the intensive care unit (F). The number at risk refers to the number of patients who were still hospitalized at the respective time after SARS-CoV-2 infection. In a total of four patients, the Omicron sublineage could not be identified.age infection
